# Efficacy of 8 Gy Single Fraction Palliative Radiation Therapy in Painful Bone Metastases: A Single Institution Experience

**DOI:** 10.7759/cureus.2036

**Published:** 2018-01-08

**Authors:** Muhammad Shuja, Ayman A Elghazaly, Asif Iqbal, Reham Mohamed, Amal Marie, Mutahir A Tunio, Moamen M Aly, Ali Balbaid, Mushabbab Asiri

**Affiliations:** 1 Department of Radiation Oncology, King Fahad Medical City, Riyadh, Saudi Arabia; 2 College of Medicine, Alfaisal University, Riyadh, Saudi Arabia; 3 Medical Physics Department, King Fahad Medical City, Riyadh, Saudi Arabia

**Keywords:** bone metastases, pain control, quality of life, radiation therapy

## Abstract

Introduction

Bone metastasis (BM) is a major complication of many solid tumors like breast, prostate, lung and renal cancers. BM leads to serious sequelae of pain, fractures, spinal cord compression and hypercalcemia. Radiotherapy has an established role in relieving pain caused by BM. Worldwide different radiotherapy schedules are being used for BM. The aim of this study is to determine the efficacy of single fraction palliative radiotherapy for painful bone metastases.

Methods

Between April 2014 and April 2017, single fraction radiotherapy was used to treat 73 patients in our institution. They had pathologically proven breast, prostate, lung or renal cancer with radiological evidence of bone metastases. There were 39 males (53%) and 34 females (47%). The median age was 58 years (range 33–87 years). 39% patients (n = 28) had breast cancer, 35% had prostate cancer (n = 26), 23% had lung cancer (n = 17), and 3% had renal cancer (n = 2). On presentation, all the patients had a pain score of more than five on Brief Pain Inventory (BPI).

Results

Response assessment to pain after three months from single fraction radiotherapy was found to be complete response (CR) in 23% patients (n = 17), partial response (PR) in 38% patients (n = 28), stable disease (SD) in 26% patients (n = 19) and progressive disease (PD) in 12% patients (n = 9). The overall efficacy of treatment was 62%, with CR 23% and PR 38%. Pre-treatment mean pain score was 8.15 compared to 4.68 post-treatment (p < 0.001).

Conclusions

Single fraction palliative radiotherapy of 8 Gy showed significant efficacy in painful bone metastases in our setting and merits further investigation in our population.

## Introduction

Many malignant diseases are known to present with bone metastases, including prostate, lung, breast and thyroid cancers [1]. Frequently involved sites of bone metastasis (BM) include vertebrae, pelvic bones, and appendicular skeleton. Although, BM often start clinically silent yet they may lead to severe sequelae as pain, fractures, spinal cord compression and hypercalcemia [2] which in turn usually have a considerable impact on the performance status and quality of life (QoL) of these patients.

Bone metastases are a frequent complication of cancer, occurring in up to 70% of patients with advanced breast or prostate cancer [3] and approximately 15 to 30% of patients with carcinoma of the lung, colon, stomach, bladder, uterus, rectum, thyroid, or kidney. The actual incidence of bone metastases is unknown, but around 350,000 people die annually because of BM in the United States alone [4]. From randomized trials in advanced breast cancer [5,6], it was observed that one of these significant skeletal events occurred on an average of every three to four months. Moreover, bone metastases may remain confined to the skeleton with succeeding decline in the quality of life, and eventually death due to skeletal and metastatic complications. After the tumors have metastasized to the bone, chances of cure are usually diminished with only 20% of breast cancer patients still alive five years after the diagnosis of bone metastases [7].

Presently, there is a greater understanding of pathophysiological mechanisms underlying the development of bony metastases, and the complex interactions and interdependence between malignant cells and bone cells [3-5]. These cancer cells, while within the bone marrow, secrete paracrine factors that harbor the capability to stimulate bone cell function. This pathological stimulation of the osteoclast or bone eating function is crucial in the osteolysis phenomena that underlie the disruption of the physiological signals between the osteoblast and the osteoclasts. This is the same mechanism that forms the basis of the use of bisphosphonates in bone metastases.

The aim of treatment in BM is pain relief, function preservation, and maintenance of skeletal integrity. Different treatment modalities are used to achieve best tumor control and delay in complication related to the metastatic bone disease. Bisphosphonates, surgical interventions, and systemic anticancer therapy are used to reach the goal. Over many years, radiotherapy (RT) has been the primary indication that proved to be useful in the treatment of painful BM [8-10].

Multiple RT schedules are in practice for palliating symptoms of BM; 30 Gy in 10 fractions over two weeks and 20 Gy in five over one week being the more commonly used regimens. However, the optimum radiation dose, fractionation regimen and treatment time remains debatable. There is increasing interest in resource sparing single fraction RT for palliating painful BM. There have been many randomized trials addressing the issue of optimal fractionation schedules and total dose of RT [11]. Regarding the end point of pain response rate assessment, many studies have shown that RT in lower doses is equivalent to higher radiation doses [12]. The use of different hypofractionation schedules other than conventional fractionation permitted obtaining variable clinical responses at better cost-effectiveness and better response rates [13]. However, the unremitting debate over the most appropriate fractionation scheme for palliative RT in bone metastases has not yet ceased.

At our center, like other major health institutions around the globe, radiation therapy services are moving towards value-based healthcare model. With the majority of patients presenting at an advanced stage disease, cost-effective, resource-sparing radiation treatment schedules are necessary to provide maximum relief. In our institute, we treat patients with single fraction RT for BM, but there is no local data available regarding its efficacy. This study will determine the effectiveness of single-fraction radiotherapy for BM in our setting and will benefit in the establishment of institutional guidelines on the use of single fraction palliative radiation therapy in the management of painful bone metastases.

## Materials and methods

Objectives

To determine the efficacy and safety of 8 Gy single fraction palliative radiotherapy in painful bone metastases in our local setting.

Study design

It was a single institution pilot study conducted at the Department of Radiation Oncology under the umbrella of Comprehensive Cancer Center at King Fahad Medical City, Riyadh, Saudi Arabia. Our study enrolled 73 patients fulfilling the eligibility criteria. Our study was conducted between April 2014 and up to April 2017. Study-specific demographic profile including age and gender information was collected on a questionnaire regarding primary disease site, and site of bone involvement.

Inclusion and exclusion criteria

The inclusion criteria included: (1) patients with age above 18 years, (2) a proven histological diagnosis of prostate, breast, lung or renal carcinoma, (3) radiological evidence of BM with (4) a pain score more than 5 on Brief Pain Inventory (BPI), (5) no previous radiotherapy to the same painful bony site, and (6) a life expectancy not less than 12 weeks. Patient performance status was used to assess the life expectancy. The study enrolled patients with functional performance status (PS) of European Cooperative Oncology Group (ECOG) 2 or less. Patients with (1) radiological evidence of pathological fractures, and (2) with spinal cord compression on magnetic resonance imaging (MRI) or computed tomography (CT) were not included in the study.

Pain assessment

Pain intensity was assessed in measurable terms by categorization on a BPI scale of 0 to 10 [14]. The score of 0 representing no pain, while the score of 10 representing the worst possible pain. Pain intensity recorded before one to two days of treatment and then three months post-treatment. Adverse effects were also noted for occurrence at the end of RT and then at three-month post-treatment follow-up visit.

Treatment and follow-up

The total dose of radiotherapy was 8 Gy in a single fraction. Treatment volume assessment was based upon the site of bone involvement. One vertebra above and one below the involved vertebrae was marked in the treatment volume in cases with spine involvement. For long bones, the treatment volume consisted of the affected region with 3-5 cm margin above and below the diseased area. The patient follow-up was at three months from the end of treatment for pain score assessment and evaluation of any late side effects.

Response assessment

Complete response and partial response (CR & PR) after three months of treatment constituted the overall efficacy. CR was taken as no pain on BPI scale on three-month post-treatment. PR was at least a 2 points decrease in pain intensity on BPI after three months from the treatment.

Data analysis

The data collected from the questionnaires were entered into the Statistical Package for Social Sciences (SPSS), version 20, software. Descriptive result for quantitative variables like age presented as mean, median with range and standard deviation (SD). Each qualitative variable like efficacy (complete and partial response), performance status, gender and site of disease presented as frequency and percentages. Comparison between the mean pain score pre-treatment and the mean pain score post-treatment at three months was calculated by paired t-test.

## Results

Patients

Among the total 73 patients enrolled in the study, 4% patients (n = 3) were of ECOG PS 0, 56% patients (n = 41) were of ECOG PS 1, and 40% patients (n = 29) were of ECOG PS 2. Gender distribution comprised of 53% male patients (n = 39), and 47% (n = 34) female patients. The mean age of the patients was 62.23 years with standard deviation 13.67; median age was 58 years (range 33–87).

A total of 39% patients (n = 28) had breast cancer, 35% (n = 26) had prostate cancer, 23% (n = 17) had primary lung cancer, and 3% (n = 2) had renal cancer (Table 1). The most common site of bone metastases was lumbar spine (34%), pelvic bone (25%), and thoracic spine (18%) (Table 2).

**Table 1:** Primary cancer frequency. Table showing frequency distribution of different cancer types included in the study.

Primary Disease	Frequency	Percentage (%)
Breast cancer	28	39%
Prostate cancer	26	35%
Lung cancer	17	23%
Renal cancer	2	3%
Total	73	100%

**Table 2:** Bone metastatic site. Table showing frequency distribution of common sites of bone metastasis.

Bone site	Number of patients	Percentage (%)
Lumbar spine	25	34
Pelvic bone	18	25
Thoracic spine	13	18
Humerus	7	10
Femur	4	6
Sternum	1	1
Cervical spine	2	2
Tibia	2	2
Clavicle	1	1
Total	73	100%

Response

At three-month post-radiotherapy, 17 patients had no pain at the disease site, so the complete response was 23%. Twenty-eight patients experienced pain improvement by 2 or more on BPI score, so the partial response was 38%. The overall efficacy was around 62% (Table 3). The pre-treatment mean pain score was 8.15 compared to 4.68 post-treatment (p < 0.001) (Table 4).

**Table 3:** Frequency distribution of efficacy. Efficacy = Complete response + partial response

Efficacy	Number of patients	Percentage (%)
Yes	45	62%
No	28	38%
Total	73	100%

**Table 4:** Mean pain score on BPI at the time of diagnosis and after radiotherapy. BPI: Bone Pain Inventory; RT: Radiotherapy.

Variables	Mean	SD
Mean BPI score from baseline	8.15	1.21
Mean BPI score after RT at three months	4.68	1.70

In our cohort, all the patients completed an uneventful three-month follow-up with no grade-3 adverse effects. A total of 11% patients (n = 8) had mild to moderate skin redness (grade II and less) which was managed with topical applications and settled within one week (four days or less).

## Discussion

In the radiation oncology clinic, BM is a commonly encountered case scenario for the attending physician. It is a typical presentation of distant relapse in a variety of malignancies including lung, breast, thyroid and prostate cancers. Bony metastases from these diverse types of cancers are often painful and affect different areas of body skeleton during their course of uninhibited growth, including the spine, pelvic bones, and bones of the extremities [15].

The primary aim of palliative radiation therapy (RT) in bone metastases is to control the pain, and additionally to preserve the normal function and maintain the integrity of the appendicular system. In the current era of modernized and advanced systemic therapies, oncology patients are expected to live longer, and similarly, they are prone to suffer from their BM for longer durations of time. Here RT offers a localized, focused and non-invasive treatment option in these cases. External beam RT is a standard and effective modality to alleviate the symptoms of pain and discomfort, with success seen in up to as much as 80% of the cases after receiving palliative radiation therapy for pain control [16].

The guidelines of National Comprehensive Cancer Network (NCCN) on cancer pain, the Ontario guidelines for palliative pain, and the third international consensus workshop on palliative radiotherapy and symptom control [17-19] all support the efficacy of palliative radiation therapy in painful bone metastases.

In 2004, Wu, et al. and the Supportive Care Guidelines Group of Cancer Care Ontario published an evidence-based practice guideline based on the results of their meta-analysis [11]. In cases with uncomplicated bone metastases where primary indication was pain relief, they recommended 8 Gy single fraction as the optimal and standard dose-fractionation schedule.

Radiation Therapy Oncology Group (RTOG) in the trial RTOG 7402 reported that shorter duration RT regimens with smaller doses were as effective as higher dose regimens for achieving pain relief in BM [20]. However, this trial was met with severe criticism for not taking into consideration several factors including use of other pain relief parameters (like narcotics) and the use of re-irradiation. While other aspects of criticism included the use of physician-based assessment tools and including a broad group of primary malignancies.

In another randomized trial by the Dutch Bone Metastasis Study group [21] published in 1999, they reported the outcomes of 1171 patients. They compared single fraction 8 Gy palliative RT to a fractionated regimen of 24 Gy in six fractions. Bone Pain Trial Working Party Study (BPTWG) [22] similarly reported their study results of 765 patients in which they randomly assigned the subjects into two arms, 8 Gy single fraction arm versus fractionated RT, which included 20 Gy in five and 30 Gy in 10 fractions. Hartsell, et al. [23] reported the outcomes of the RTOG trial 9701, which compared the efficacy of short-term RT (8 Gy single fraction) versus long-course RT (30 Gy in 10 fractions) for treating painful BM. All these trials concluded that single fraction RT was equally efficacious to protracted regimen RT schedules of comparatively longer durations regarding sustained response to pain. The results of our study were in concordance with similar findings of single-fraction RT with 8 Gy as an effective regimen to achieve a response in painful bony metastases.

In RTOG 9701, eligibility criteria included the patients with breast or prostate cancer only, with an expected survival of three months or greater. This study provided a homogeneous group of patients whose potential for long-term survival would allow for sufficient follow-up in order to ascertain the long-lasting effect of the two treatments. This review appeared in contradiction to the Dutch Bone Metastases trial and the BPTWG, which targeted bony metastases from various primary malignancies and did not include prognosis in the early eligibility criteria. In our study, we evaluated patients with histological diagnosis of breast, prostate, lung and renal cancer. Our inclusion criteria included patients with an expected survival of at least 12 weeks to allow us a follow-up pain score assessment on three-month post-treatment visit.

In the RTOG 9701 trial, there were no differences in the CR and PR rates between the two arms of the RTOG 9701 trial. CR was 17% in the 30 Gy long-term arm versus 15% in the 8 Gy single fraction short-term RT arm, while PR was 49% versus 50%, respectively. In the Dutch and BPTWG trials, there were also no differences in response rates between the single-fraction arm compared to the one with protracted RT schedules. In the Dutch study, there was a 71% overall response (with at least 2 points decrease on the pain scale), whereas the complete response rate was 35% (no pain, and independent of analgesic requirement). In the BPTWG trial, the reported overall response was 78%, with a 57% complete response rate (no pain, and independent of analgesic requirement). Regarding primary endpoints, 8 Gy single fraction RT in our cohort was able to achieve a 62% overall response rate, with 23% complete response and 38% partial response. It was found similar to other trials comparing single fraction RT to more extended RT schedules.

In our study, we included patients with primary breast, prostate, lung, and renal carcinoma. The most common primary malignancy was breast cancer (39%) followed by prostate cancer (35%). The most common site of bony metastasis was found to be the lumbar spine with 34% cases (n = 25) followed by pelvic bone in 25% cases (n = 18).

Our study yielded a 62% overall response rate with 23% achieving a complete response. The mean pain score pre-treatment was 8.15 compared to 4.68 post-treatment (p < 0.001). Results of our study and other randomized trials, strongly propose the use of single fraction palliative RT as an effective treatment in patients with painful BM. In our study, we did not experience any grade 3 toxicity, either immediately after the treatment or at three-month post-treatment follow-up. Eleven percent patients (n = 8) had mild to moderate skin redness (grade II and less) which was managed with topical steroidal applications and settled within one week (four days or less).

Our study was a single arm prospective pilot project undertaken to evaluate the scope of 8 Gy singular fraction palliative RT in our setting. Our study limitations include smaller sample size, single-center experience and the inclusion of specific cancer types. Hopefully, a well-designed double-arm randomized trial taking into consideration all cancer subtypes and comparison to different RT regimens would be ideal to better inform on the outcomes of single fraction versus more extended RT regimens.

## Conclusions

8 Gy single fraction palliative radiotherapy for the painful bone metastases appears to provide equivalent efficacy for pain control compared to protracted regimens. Our response rate of 62% is parallel to the international published data from randomized trials. Single fraction regimen may be considered as more practical and cost-effective in situations where the radiation treatment facilities are limited in regards to the number of patients.
